# Late diagnosis of Lesch–Nyhan disease complicated with end-stage renal disease and tophi burst: a case report

**DOI:** 10.1080/0886022X.2020.1713805

**Published:** 2020-01-27

**Authors:** Cahyani Gita Ambarsari, Daffodilone Cahyadi, Lenny Sari, Oryza Satria, Felly Sahli, Thyrza Laudamy Darmadi, Agustina Kadaristiana

**Affiliations:** a Pediatric Nephrology Division, Department of Child Health, Faculty of Medicine, Cipto Mangunkusumo Hospital, University of Indonesia, Central Jakarta, Indonesia; b Pediatric Centre, Pondok Indah Bintaro Jaya Hospital, South Tangerang, Indonesia; c Orthopaedic Centre, Pondok Indah Bintaro Jaya Hospital, South Tangerang, Indonesia; d Pathology Anatomy, Pondok Indah Bintaro Jaya Hospital, South Tangerang, Indonesia; e Department of Orthopaedic and Traumatology, Fatmawati Hospital, South Jakarta, Indonesia; f Radiology Centre, Pondok Indah Bintaro Jaya Hospital, South Tangerang, Indonesia; g Laboratory Unit, Pondok Indah Bintaro Jaya Hospital, South Tangerang, Indonesia

**Keywords:** Lesch–Nyhan syndrome, hypoxanthine, phosphoribosyltransferase, hyperuricemia, gout, chronic kidney failure, case report

## Abstract

**Background:**

Lesch–Nyhan disease (LND) is a rare X-linked recessive inborn error of purine metabolism. Late diagnosis of LND may cause significant morbidity. LND cases have never been reported in Indonesia.

**Case report:**

A 15-year-old male who had been diagnosed with cerebral palsy was referred to our hospital due to renal failure requiring emergency dialysis. The patient presented with three classic manifestations of LND: increased uric acid levels, neurological disorders, and self-injurious behaviors. LND was suspected because of an abscess-like lump on the left ankle that was confirmed to be a tophus, which had burst and discharged thick masses containing blood, debris, and white crystal materials. The diagnosis of LND was confirmed by the presence of a deletion to exon 1 of the HPRT1 gene. The patient received oral allopurinol daily and treatment for end-stage renal disease (ESRD), which included regular dialysis and subcutaneous administration of erythropoietin. At a 2-month follow-up, he improved clinically with a 71% decrease in uric acid levels after regular dialysis and allopurinol treatment.

**Conclusion:**

In developed countries, LND can be diagnosed as early as 3 days after birth. However, diagnosis in the present case was delayed due to the rarity of the disease and the limited number of facilities in Indonesia that offer genetic counseling. Late diagnosis of LND leads to ESRD and irreversible abnormalities. This is the first case of LND presenting with a unique clinical presentation of tophus burst reported in Indonesia.

## Background

Lesch–Nyhan disease (LND, OMIM #300322) is the most severe manifestation of hypoxanthine-guanine phosphoribosyltransferase (HPRT) enzyme deficiency, which is characterized by overproduction of uric acid, neurological disorders, and behavioral problems [1,2]. LND is a rare X-linked recessive genetic disorder (Xq26.2–q26.3) that commonly affects males [3]. In LND, mutations to the HPRT1 gene result in diminished activity of HPRT, an enzyme that plays a central role in the generation of purine nucleotides in the purine salvage pathway, resulting in increased uric acid levels [1]. Although first recognized in 1964, LND is commonly misdiagnosed, as patients with LND are assumed to have early-onset cerebral palsy during the course of the disease [2,4]. Late diagnosis of LND may result in severe morbidity, as with the present case. Here, we report a male who was diagnosed with LND at the age of 15 years in Indonesia. LND cases have never been reported in Indonesia. This case is probably the second report of LND with chronic renal failure. Notably, this case report provides detailed information about the clinical manifestations of LND, such as spontaneous tophi bursts.

## Case presentation

A 15-year-old male with renal failure was referred to our center for dialysis treatment. Two weeks prior to referral, he was brought to an orthopedist for a suspected bone disorder characterized by worsening lumps on his left hand and ankle, which was noticed by his mother about 1 year before presentation. However, she had assumed that the swelling to his left hand was due to biting. In addition, swelling to his left ankle occurred after several leg strengthening exercises conducted by a physiotherapist. The orthopedist later recommended magnetic resonance imaging (MRI). On MRI preparation, the patient was found to have a high urea level and was thus referred to a pediatric nephrologist who subsequently initiated dialysis. His mother also claimed that the urine volume of her son had decreased within the last few weeks. On the second day of hospitalization, the swelling in his left ankle burst and thick masses containing blood, debris, and white crystalized materials were observed.

**Figure 1.**
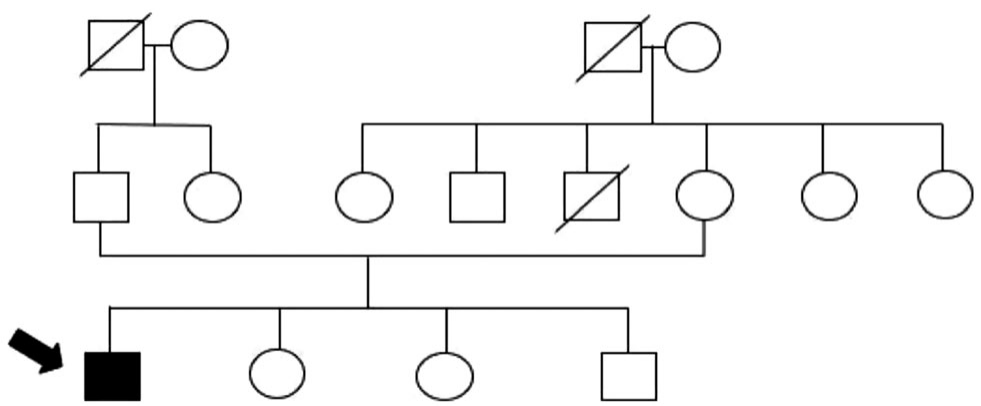
Family tree.

The patient was born to healthy non-consanguineous parents with no family history of HPRT deficiency (Figure 1). At the age of 1 day, the patient developed stiffness in both hands. When he was 2 months old, he was diagnosed with severe psychomotor retardation and could only lie down. A pediatric neurologist diagnosed the patient with cerebral palsy at the age of 7 months based on computed tomography results showing cerebral atrophy. The patient has a habit of biting his lower lip, buccal mucosa, and left hand, which was evident since the age of 6 years, for which an occlusal bite guard was once used years before to protect his teeth. However, he refused to use it, which resulted in the extraction of some of his upper teeth. At present, he is unable to talk or communicate normally, but can express anger through agitating behavior. Moreover, his parents remarked about the occasional presence of orange sand-like sediments found in his diaper, which first occurred at the age of 3 years. However, a local pediatrician assumed that the patient was merely dehydrated and recommended increased water intake and intravenous fluid treatment, but neglected to measure his uric acid levels.

**Figure 2.**
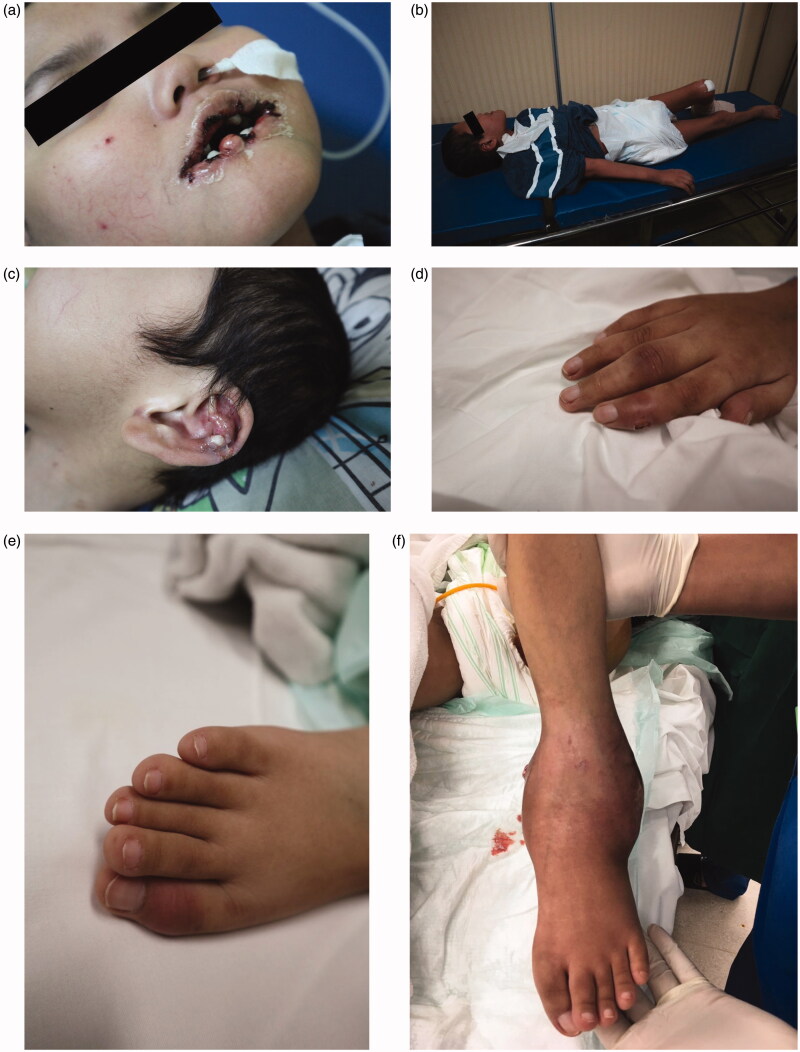
(a) Laceration to the lower lip due to self-mutilation behavior. (b) Inability to stand or walk, could only lie down. (c) Tophus on the antihelix of the earlobe. (d) Tophus of the right hand. (e) Tophus of the right foot. (f) Tophus and discharging wound of the left ankle.

On a physical examination, deformities to the lower lip were noted (Figure 2(a)). A neurological examination revealed hypotonicity, dystonia, and clonus of all four extremities (Figure 2(b)). Single lump was seen on the antihelix of the earlobe (Figure 2(c)), along with multiple lumps on his extremities (Figure 2(d,e)) and larger lumps similar to abscesses on his left hand and ankle (Figure 2(f)). He had scoliosis and flexion contracture of the hip, knee, and shoulder. He weighed 22 kg and was 140 cm tall (less than the third percentile of the Centers for Disease Control and Prevention, National Center for Health Statistics 2000 growth chart). The diuresis rate was 0.9 mL/kg/h. The laboratory workup results showed a hemoglobin level of 60 g/L (6.0 g/dL), serum uric acid level of 1608 µmol/L (27 mg/dL), urea level of 99.96 mmol/L (280 mg/dL), creatinine level of 671.8 µmol/L (7.59 mg/dL), and an estimated glomerular filtration rate of 7.6 mL/min/1.73 m^2^ (New Schwartz).

24-h urine collection revealed a uric acid level of 1.1 mmol/24 h (186 mg/24 h) (with a total urine volume of 430 mL) and random urine uric acid to creatinine (UA/C) ratio of 2.7 (mg/mg). Electrolyte analysis indicated metabolic acidosis (bicarbonate level of 14.1 mmol/L), hyponatremia (sodium level of 129 mmol/L), hyperkalemia (potassium level of 7.14 mmol/L), and hyperphosphatemia (2.77 mmol/L). Other electrolytes were within normal limits.

**Figure 3.**
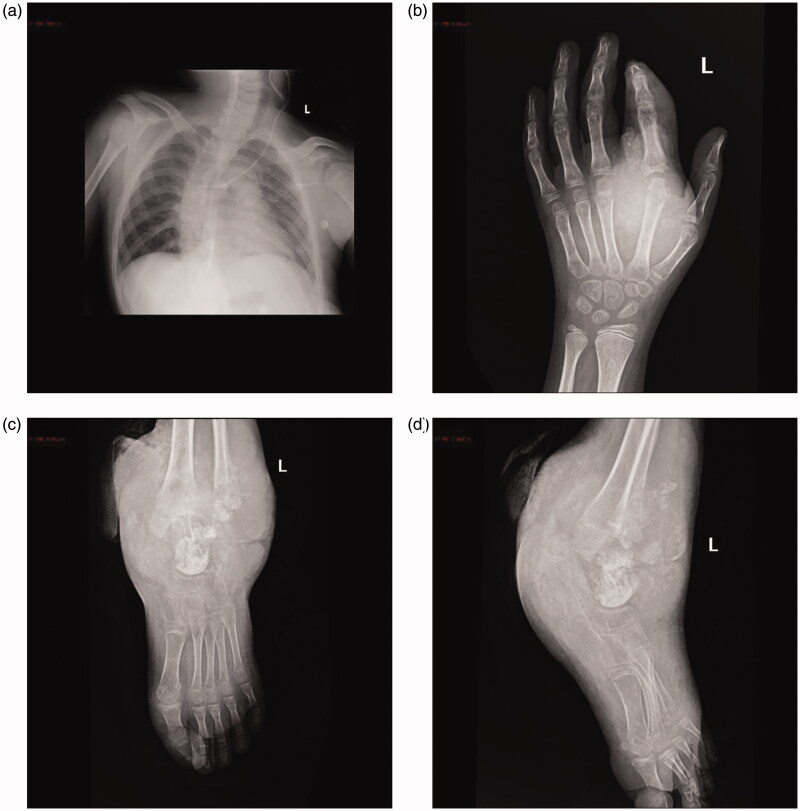
(a) Chest radiography showing thoracic scoliosis (anteroposterior and supine views). (b) X-ray image of the left wrist showing a soft tissue mass adjacent to the metacarpal and proximal to the second finger. (c) X-ray image of the left ankle showing a large soft tissue mass with intramass calcification.

**Figure 4.**
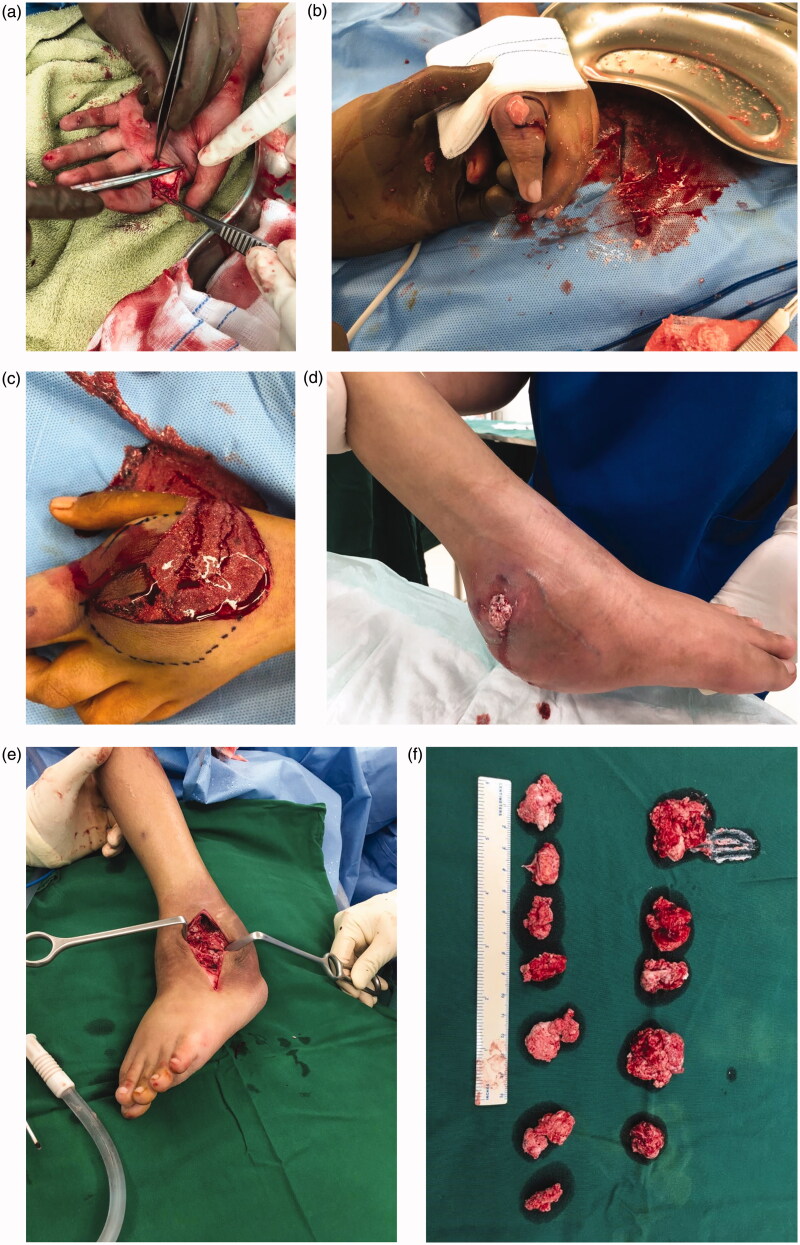
(a–c) Tophus of the left index finger and wrist. (d–f) Tophus of the left ankle burst and discharged stones and crystals.

**Figure 5.**
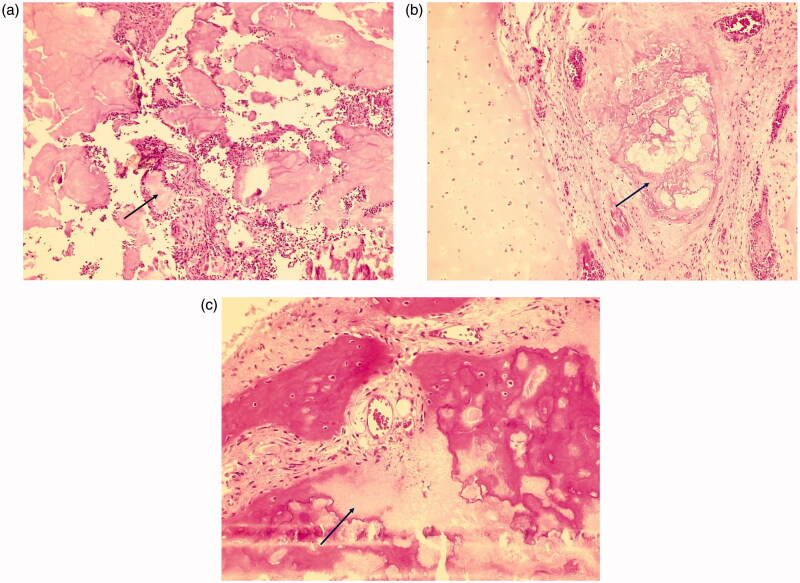
(a and b) Bone biopsy revealed the presence of urate crystals (hematoxylin and eosin staining, ×100 original magnification) and (c) demineralization (hematoxylin and eosin staining, ×100 original magnification).

Chest radiography showed thoracic scoliosis (Figure 3(a)). Arthrography or joint X-ray of the left hand and ankle showed revealed the presence of tissue masses (Figure 3(b–d)). After the lump on the left ankle burst, debridement and exploration sessions were performed in the left wrist and ankle, which revealed tophi of several joints (Figure 4(a–f)). Along with the debridement and exploration procedures, bone biopsy results confirmed juvenile gouty arthritis (Figure 5(a–c)).

**Figure 6.**
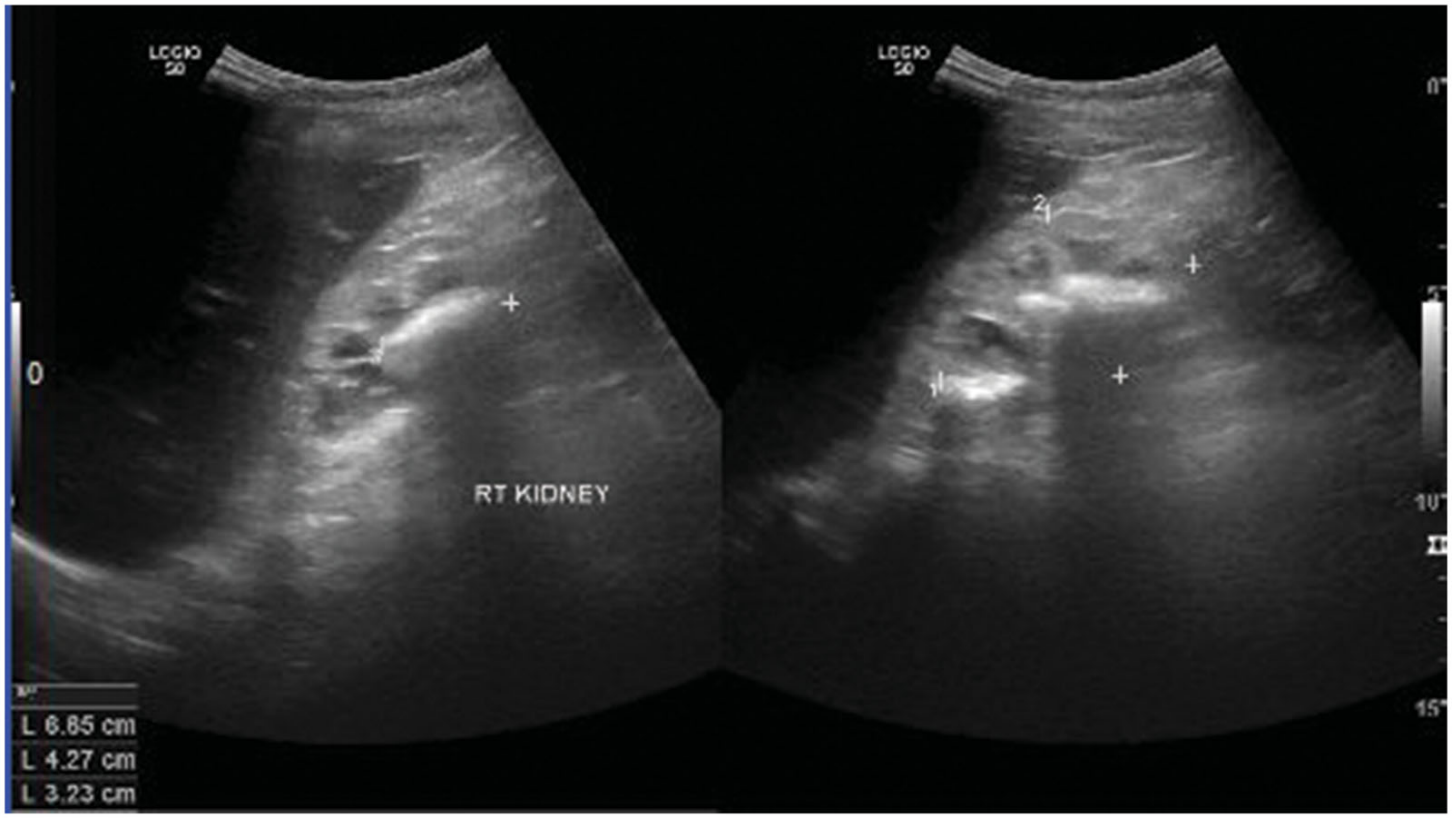
Multiple stones in the right renal pelvis.

Kidney ultrasonography revealed bilateral atrophic kidneys (left kidney: 5.4 × 2.9 cm; right kidney: 6.9 × 4.3 cm) with multiple stones in the right renal pelvis (Figure 6). A diagnosis of LND was confirmed *via* genetic testing showing a homozygous deletion of exon 1 of the HPRT1 gene. The samples were sent to a foreign genetic laboratory and the results were obtained 1 month later. HPRT enzyme levels could not be measured in a timely fashion due to logistic challenges, such as unstable temperature for sample delivery and the inability to provide a short delivery time.

Hemodialysis was initiated, which was followed by regular hemodialysis within a week, but was switched to continuous ambulatory peritoneal dialysis to provide better long-term quality of life for the patient and his caregiver. The patient received a red blood cell transfusion, allopurinol treatment at a dose of 300 mg/day, a low-purine diet, and treatment for ESRD, which included subcutaneous erythropoietin (EPO) supplementation of 6000 U/week, oral calcitriol at a dose of 0.5 mcg daily, oral CaCO_3_ at 500 mg three times daily (with a meal), oral folic acid at 5 mg once daily, and one tablet of oral vitamin B complex daily. With treatment, serum uric acid levels decreased to 470 µmol/L (7.91 mg/dL) within 2 weeks. During a follow-up visit at 2 months after the tophi burst, the patient appeared to be more responsive when communicating with others. Moreover, the swelling to his ankle and wrist had significantly improved. The patient complied well to the treatment regimen and peritoneal dialysis caused no side effects. The results of a follow-up laboratory workup at the outpatient clinic were as follows: serum uric acid level of 336 µmol/L (5.65 mg/dL), serum urea level of 14.2 mmol/L (39.76 mg/dL), serum creatinine level of 323 µmol/L (3.65 mg/dL), and random urine UA/C ratio of 0.42 mg/mg. The parents were advised to undergo genetic consultation to identify if their daughters were carriers or if their other son also carried the mutation. The prognosis of the patient was poor because of the presence of ESRD accompanied with multiple irreversible abnormalities.

## Discussion

Here, we present a case of a 15-year-old male with extreme presentations of ESRD and tophi burst with a late diagnosis of LND. Misdiagnosis of LND is common due to the rarity of the disease. The prevalence of LND in the United Kingdom is 1 case per 2 000 000 persons with an incidence of 1/500 000 live births [5]. However, the incidence of LND in Indonesia remains unclear and no LND cases were reported before. Furthermore, the symptoms of LND, such as motor retardation and cognitive disorder, are similar to those of cerebral palsy [2,6], as with the present case.

LND is the most severe manifestation of HPRT deficiency. The spectrum of the clinical manifestations of HPRT deficiency varies depending on enzymatic activity. In LND, motor delay is accompanied by uric acid overproduction and behavioral disorders, as with the present case. Recurrent self-injurious behaviors (SIBs), such as biting of the fingers, hands, lips, and cheek, which can be accompanied with banging of head or legs, are hallmarks of LND. SIBs usually occur between the ages of 2 and 3 years, but almost always before the age of 4 years. Nevertheless, the onset of SIBs can be delayed up to the age of 20 years, which could delay or complicate a diagnosis of LND [7]. In partial HPRT deficiency (Kelly-Seegmiller syndrome), only uric acid overproduction occurs without neurological involvement [8]. Phosphoribosylpyrophosphate synthetase superactivity also presents with hyperuricemia, kidney stone, and neurological symptoms, however it exhibits hearing impairment, differing it from HPRT deficiency [9].

In this case, the patient had to undergo emergency dialysis due to renal failure. It is known that increase of 1 mg/dL uric acid level from baseline escalates the risk of CKD progression by 28% [10]. Since the age of 3 years, our patient intermittently experienced excretion of sand-like urine, which is actually the early sign of uric acid overproduction. The manifestation of sand-like urine in this patient, which was characterized by orange sand-like sediments found in his diapers, had likely occurred later as compared with other cases and typically presents within the first year of life [11]. A LND population study in UK showed that there were 8/23 (34.8%) LND patients who reported crystals in diapers [5]. However, in the present case, this manifestation was considered as dehydration. Consequently, the overproduction of uric acid was underdiagnosed, thus the patient did not receive appropriate treatment. Measurement of urine uric acid/creatinine ratio in children with delayed development is proposed recently to allow early detection of LND [12].

As a result, uric acid in the blood was then accumulated in the tissue and manifested as tophaceous gout [8,13]. Similar to this case, a case of delayed diagnosis of LND was reported; upon which gouty tophus had been exhibited before the final diagnosis was made [14]. Repetitive trauma from the habit of hand biting and improper exercise during physiotherapy may exacerbate gout attacks. In this case, gout attacks were characterized by intense swelling, pain, and tophi burst [15]. Cases of tophus burst in chronic gout in elderly were reported at serum uric acid level of 10.2 mg/dL (606.7 µmol/L), 532 µmol/L, and 8.8 mg/dL (523.42 µmol/L); much lower compared to our patient [16–18].

To compensate for hyperuricemia, the excretion of uric acid is increased in the kidneys, which may increase the risk of developing uric acid stones in the kidneys and urinary tract [13]. Since the development of allopurinol, a purine analog, ESRD due to HPRT is rarely observed in developed countries, which explained low incidence of renal disease in LND these days [2]. However, this condition can still be occurred if diagnosis is delayed. In our patient, ESRD, which is characterized by stunted growth, severe anemia, and mineral bone disease, might have been caused by hyperuricosuria, nephropathy, and obstructive uropathy due to the formation of uric acid calculi, as shown by the kidney ultrasonography results in the present case, and random increases of the urine UA/C ratio [19,20].

Laboratory workup plays a vital role in establishing the diagnosis of LND. A random urine uric acid/creatinine (UA/C) ratio of >2 is a characteristic LND patients aged <10 years [2]. Other laboratory workup results indicative of LND include a 24-h urinary uric excretion of >20 mg/kg and serum uric acid level of >8 mg/dL (hyperuricemia) [2]. The random urine UA/C ratio of our patient was 2.7 mg/mg and he also had hyperuricemia with a serum uric acid level of 1608 µmol/L (27 mg/dL). Unfortunately, measurements of the random urine UA/C ratio, serum uric acid level, and 24-h urinary uric acid excretion are not sufficient to establish a diagnosis of LND because of the low sensitivity and specificity of analyses [2]. The 24-h urinary uric excretion value should be cautiously interpreted because bacterial contamination or uric acid precipitation during sample collection hinders accurate measurement [2]. Utilization of urine sample for this purpose should be done when it is fresh, otherwise it would need rewarming to prevent precipitation [21]. Our case had normal 24-h urinary uric acid excretion and abnormal UA/C, implying that 24-h urinary uric acid excretion and random urine UA/C ratio would be very difficult to interpret in the face of severe renal disease. The diagnosis of LND can be confirmed by evaluating HPRT enzyme activity and the proliferation of peripheral blood T-lymphocytes or genetic testing [2].

Genetic testing of our patient revealed a deletion to exon 1 of the HPRT1 gene, which has been reported in several cases [22]. Deletion of the coding region of the HPRT1 enzyme is associated with a severe phenotype of LND, since the structure of the protein is abnormal and with no enzymatic activity [23].

LND treatment aims to reduce uric acid production, alleviate neurological symptoms, and prevent further morbidities due to SIBs [2]. Our patient was treated with 300 mg of allopurinol daily, an effective and safe drug that reduces uric acid production in patients with LND [2,24]. The uric acid serum level can be reduced to approximately 47% and the random urine UA/C ratio can be decreased by 74% or more within 12 months [24]. In our patient, the serum uric acid level had decreased as much as 71% within 2 weeks of treatment. In addition to allopurinol treatment, the rapid decrease in serum uric acid level may be attributed to dialysis. Peritoneal dialysis was preferred due to sustain clearance and ultrafiltration [25]. Average serum uric acid in patients underwent peritoneal dialysis is 7.7 mg/dL (458 µmol/L) [26], while in our patient it was 336 µmol/L after initiation of peritoneal dialysis. Although allopurinol is effective, it should be used with caution to avoid increased excretion rates of hypoxanthine and xanthine, which are reported to increase by 5–10 times, as compared to baseline levels [24]. As a result, xanthine accumulation and lithiasis may occur. Therefore, adjustment of the allopurinol dosage is essential to achieve optimal uric acid levels without experiencing side effects, such as xanthine accumulation. Unlike increased uric acid levels, neurological and behavioral disorders cannot be optimally managed [27]. Regardless of allopurinol treatment, LND patients still present with behavioral problems, indicating that behavioral problems are not associated with increased uric acid levels [28]. SIBs are managed by physical protection, behavioral intervention, and medications [29]. Unfortunately, these measures can only partially control such SIBs [27].

The prognosis of LND is dependent on the how fast the diagnosis can be made and the treatment provided. In developed countries, LND can be diagnosed as early as 3 days after birth [20]. Early diagnosis of LND is essential to provide immediate treatment and genetic counseling is advised to prevent LND-related birth defects in future pregnancies [5]. With appropriate allopurinol treatment, patients with LND can survive up to the second or third decade of life [2]. Allopurinol can also provide long-term protection to the kidneys [5].

Unfortunately, the prognosis of our patient was poor due to the delayed diagnosis. Our patient will probably be highly dependent on other individuals and the use of a wheelchair due to severe motor retardation. Prior LND cases with dialysis were reported at 4 and 4.5 years of age [30,31], indicating that this case is long overdue. In those cases, peritoneal dialysis was not the initial treatment, but later it was started due to progressing renal insufficiency; while in our case, the initial GFR was already 7.6 mL/min/1.73 m^2^ at the age of 15 years old. Besides, the late diagnosis in this case was likely due to the rarity of LND in Indonesia. Furthermore, the clinical symptoms of our patient were similar to those of cerebral palsy, and no facilities in Indonesia offer genetic testing facility and assessment of the HPRT enzyme. Although the presence of of SIBs can help to differentiate LND from cerebral palsy, the development of LND is usually delayed and, therefore, cannot be used as a standard for early diagnosis [7].

The present case is unique, as this may be the first reported case of LND associated with a tophus burst. Recognition of this disease is important to provide prompt diagnosis and treatment, therefore further complications could be prevented. In addition, early signs of uric acid overproduction, such as orange sand-like sediments in diapers and hyperuricemia in patients with motor delays, should raise a suspicion of LND.

## Conclusion

Our patient presented with classical features of LND. In developed countries, LND can be diagnosed as early as 3 days after birth. However, diagnosis of the present case was delayed because of the rarity of the disease and the limited number of facilities in Indonesia that offer genetic counseling. A late diagnosis of ESRD leads to irreversible abnormalities.
